# Sequential versus upfront oxaliplatin-based therapy in metastatic colorectal cancer: long-term outcomes of a randomized phase 3 trial

**DOI:** 10.1038/s43856-026-01633-3

**Published:** 2026-05-08

**Authors:** Makoto Okawaki, Mototsugu Shimokawa, Ryo Inada, Hitoshi Ojima, Hiroaki Tanioka, Shingo Noura, Yoshinori Munemoto, Keiichiro Ishibashi, Yoshiaki Shindo, Madoka Hamada, Masasumi Okajima, Yoshiyuki Yamaguchi, Takeshi Yamada, Yasuhiro Shimada, Takeshi Nagasaka

**Affiliations:** 1 Department of Clinical Oncology, Kawasaki Medical School Hospital, Kurashiki, Okayama Japan; 2 Department of Biostatistics, Yamaguchi University Graduate School of Medicine, Ube, Yamaguchi Japan; 3 Department of Gastroenterological Surgery, Kochi Health Sciences Centre, Kochi, Kochi, Japan; 4 Department of Gastroenterological Surgery, Gunma Prefectural Cancer Centre, Ota, Gunma Japan; 5 Department of Surgery, Osaka Rosai Hospital, Sakai, Osaka Japan; 6 Department of Surgery, Fukui-ken Saiseikai Hospital, Fukui, Fukui, Japan; 7 Department of Digestive Tract and General Surgery, Saitama Medical Centre, Saitama Medical University, Kawagoe, Saitama Japan; 8 Department of Gastroenterological Surgery, Nakadori General Hospital, Akita, Akita, Japan; 9 Department of Gastrointestinal Surgery, Kansai Medical University Hospital, Hirakata, Osaka Japan; 10 Department of Surgery, Hiroshima City Hospital, Hiroshima, Hiroshima, Japan; 11 Department of Gastroenterological Surgery, Nippon Medical School, Tokyo, Japan; 12 Division of Clinical Oncology, Kochi Health Sciences Centre, Kochi, Kochi, Japan

**Keywords:** Chemotherapy, Phase III trials

## Abstract

**Background:**

Whether oxaliplatin should be given sequentially or upfront in metastatic colorectal cancer remains clinically relevant, especially for older patients often underrepresented in trials.

**Methods:**

We report updated overall survival and prespecified quality-of-life outcomes from the open-label, randomized, multicenter phase 3 C-cubed trial in Japan. This trial was registered in the University Hospital Medical Information Network Clinical Trials Registry UMIN000015405 (October 14, 2014) and UMIN000036690 (May 10, 2019). The primary endpoint was reported previously. Of 311 randomized patients with previously untreated metastatic colorectal cancer, 300 were included in the full analysis set. Patients received fluoropyrimidine plus bevacizumab, with oxaliplatin added at progression, or received fluoropyrimidine, oxaliplatin, and bevacizumab from the start. Overall survival was analyzed using Cox regression and time-restricted survival methods. Quality of life was assessed prospectively using patient-reported questionnaires.

**Results:**

Here we show that overall survival is similar between strategies. Median overall survival is 27.2 months with sequential treatment and 27.4 months with upfront treatment (hazard ratio, 1.00; 95% confidence interval, 0.76–1.33; p = 0.98). Additional time-restricted and milestone analyses show no between-group differences. There is no evidence that age modifies the relative treatment effect. Patient-reported outcomes indicate a lower early treatment burden with sequential treatment, including smaller early declines in physical functioning and less sensory neuropathy.

**Conclusions:**

Updated overall survival is comparable between sequential and upfront oxaliplatin-based strategies, while sequential treatment is associated with lower early treatment burden. These findings support individualized selection of first-line treatment based on age, vulnerability, and treatment goals.

## Plain language summary

Colorectal cancer is common, particularly in older adults. The standard treatment, chemotherapy, often combines several drugs, which can cause serious side effects, such as nerve damage, which can reduce quality of life. We conducted a clinical trial to compare two treatment strategies. In one group, people were given drugs that were less likely to cause severe side effects, fluoropyrimidine plus bevacizumab. A drug more likely to cause severe side effects, oxaliplatin, was only added if cancer progressed. The other group started with all three drugs simultaneously. The long-term survival of people in the two groups was similar but those who were given the oxaliplatin later experienced fewer side effects and had a better quality of life. Thus using a sequential treatment strategy might improve the quality of life for people with cancer.

## Abstract (web summary)

Okawaki et al. report updated overall survival and quality-of-life results from a randomized phase III trial comparing sequential versus upfront oxaliplatin-based therapy in metastatic colorectal cancer. They show comparable overall survival, with lower early treatment burden and less neuropathy with the sequential strategy.

## Introduction

Colorectal cancer (CRC) is one of the most frequently diagnosed cancers worldwide, particularly in Western countries and Japan, and its incidence rises sharply with age^1,2^. This demographic trend is clinically important because, in cohorts and trials of advanced CRC treated with combination chemotherapy, older age may be associated with less favorable outcomes, underscoring the need to optimize treatment strategies for older adults^3,4^.

The current standard of care for metastatic CRC (mCRC) typically involves comprehensive chemotherapy regimens, such as doublet or triplet therapies, in combination with monoclonal antibodies.^5^ Treatment strategies are guided by patient-specific factors, including performance status (PS), molecular profiles (e.g., *RAS* and *BRAF* mutations), and the primary tumor’s location. Tumor location, mainly whether the primary tumor arises in the right colon or left colon, has been recognized as a critical factor influencing treatment outcomes^6,7^. Right-sided tumors often demonstrate distinct biological behaviors, including higher rates of *RAS* and *BRAF* mutations, and are associated with worse prognoses compared to left-sided tumors^7,8^. However, the mechanisms underlying these differences and their implications for treatment strategies remain areas of ongoing investigation.^9^

Despite advances in treatment, there remains a significant gap in clinical research; most clinical trials predominantly include patients under the age of 65, leaving the elderly population underrepresented^10^. Emerging studies have begun to evaluate alternative therapeutic approaches, including fluoropyrimidine (FP) combined with bevacizumab (BEV) and dose-adjusted chemotherapy, as viable options for older patients^11^.

The XELAVIRI study (AIO KRK-0110) offered valuable insights into the treatment of mCRC by comparing two regimens: an initial therapy of FP and BEV followed by stepwise escalation to irinotecan, versus a simultaneous combination of FP, irinotecan, and BEV from the start^12^. Notably, the study revealed that, particularly for patients older than 75, upfront combination therapy did not yield significant improvements in outcomes compared to a sequential treatment approach^13^. Building on these findings, the C-cubed study investigates two treatment strategies for mCRC: one that initiates therapy with FP and BEV followed by escalation to oxaliplatin (OX) and FP (Arm A), and another that starts with a combination of FP, OX, and BEV (Arm B)^14^.

In this updated analysis of the randomized phase III C-cubed trial, we show that sequential FP plus BEV with deferred OX achieves overall survival (OS) comparable to upfront OX-based combination therapy, with lower early treatment burden. Tumor location and *RAS/BRAF* mutation status remain major prognostic factors, and patient-reported outcomes show smaller early declines in physical functioning and less sensory neuropathy with the sequential strategy. These findings support a patient-centered, age- and vulnerability-adapted approach to first-line treatment selection in mCRC.

## Methods

### Study design and conduct

This open-label, randomized, multicenter phase III trial evaluated two treatment strategies for metastatic colorectal cancer (mCRC). A total of 311 patients were randomized 1:1 into Arm A (sequential treatment: FP plus BEV with escalation to OX upon progression) or Arm B (upfront combination therapy: OX, FP, and BEV); the full analysis set comprised 300 patients. Patients were allocated across 81 Japanese institutions, with stratification by critical variables including study site, Köhne Index (low/intermediate/high), and prior adjuvant chemotherapy (with or without OX)^15,16^. The primary endpoint, time to failure strategy (TFS), was previously reported^14^. Patients were accrued in the original C-cubed trial between December 2014 and September 2016 across 81 participating institutions in Japan. This update focuses on overall survival, with follow-up completed by the data cutoff date (31 July 2020).

### Patients

Baseline demographic and clinical data were collected, including age, sex, Eastern Cooperative Oncology Group (ECOG) performance status (PS), tumor characteristics, laboratory parameters, and prior treatments. Patients were categorized by age into <70 years and ≥70 years.

### Treatment

Crossover administration between capecitabine and 5-fluorouracil (5-FU) was not permitted.

#### Arm A (Sequential treatment)

Patients received BEV plus either capecitabine or 5-FU, as determined by the treating physician, until radiologic progression; after progression, OX was added within 1 month.

#### Arm B (Upfront combination treatment)

Patients received upfront treatment with OX, BEV, and either capecitabine or 5-FU, as determined by the treating physician. After 12 weeks or upon OX-associated toxicity, treatment was de-escalated to capecitabine or 5-FU plus BEV. Re-escalation was permitted only for patients who had de-escalated OX due to the treatment schedule rather than toxicity.

### Efficacy endpoints and toxicity assessments

Tumor response was assessed every eight weeks using computed tomography (CT) or magnetic resonance imaging (MRI), based on the response evaluation criteria in solid tumors (RECIST) version 1.1. The primary endpoint was previously published^14^, while this analysis focuses on secondary endpoints, including OS and the discovery of prognostic factors. Adverse events were graded according to the National Cancer Institute Common Terminology Criteria for Adverse Events (CTCAE) version 4.0.

### RAS and BRAF mutation detection

*RAS* mutations (*KRAS* and *NRAS*) were assessed in 286 participants. Among these, 83 in Arm A and 85 in Arm B carried *RAS* mutations. For *RAS* wild‑type (*n* = 118), *BRAF* V600E mutations were evaluated by Sanger sequencing (codon 600)^14^.

### QoL assessments

Quality-of-life (QoL) was prospectively assessed at baseline, 6, 12, and 18 months using the EORTC QLQ‑C30, EQ‑5D‑3L, and the Patient Neurotoxicity Questionnaire (PNQ). Analyses were prespecified in the protocol. For QLQ‑C30, domain‑specific change scores were calculated as Δ = follow‑up − baseline; for functional scales, a negative Δ indicates deterioration, and for symptom scales, a positive Δ indicates worsening. For the EQ-5D-3L, we analyzed both the index value at each visit and the change from baseline (Δ, as above). The percent change from baseline was calculated as the mean Δ divided by the baseline mean within each arm. PNQ sensory and motor severities were scored on a 0–4 ordinal scale (responses were recorded as 1–5 and recoded to 0–4 for presentation), with PNQ Δ defined as follow‑up − baseline. Between‑arm comparisons of QLQ‑C30 change scores, EQ‑5D‑3L index change, and PNQ Δ at each time point were prespecified as exploratory and performed with the Wilcoxon rank‑sum test without multiplicity adjustment. Missing questionnaires were not imputed; the sample size at each visit reflects the number of respondents.

### Statistics and reproducibility

**Fig. 1:**
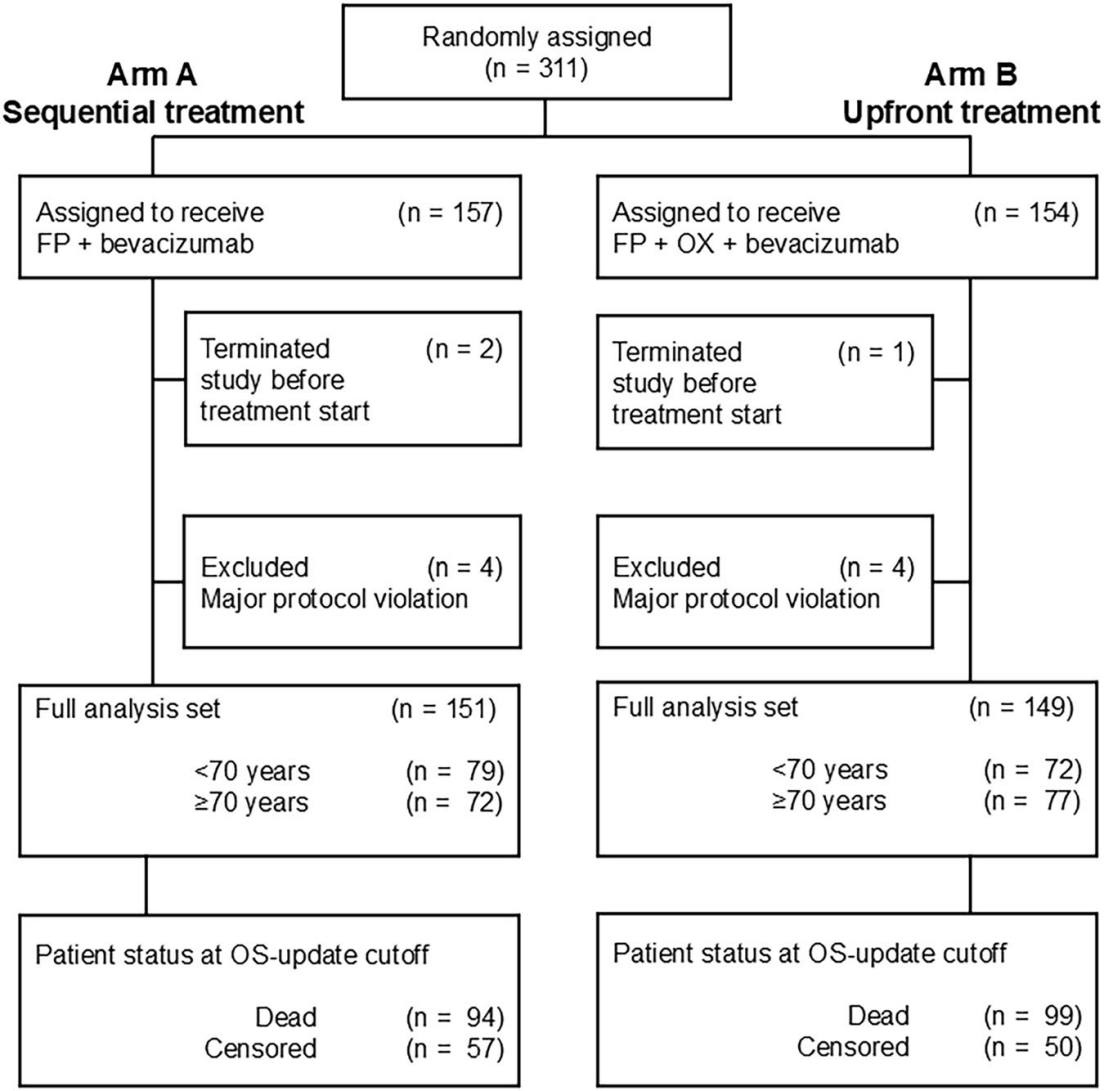
Flow diagram of patients included in the updated overall survival (OS) analysis. A total of 311 patients were randomized; after excluding those who did not initiate treatment or had major protocol violations, 300 patients were included in the full analysis set, stratified by treatment arm and age group (<70 vs. ≥70 years).

The full analysis set (FAS, modified intention-to-treat population) comprised 300 patients after excluding those who did not initiate treatment (*n* = 3) and those with major protocol violations (*n* = 8) (Fig. 1). All analyses were performed in JMP 18.2.0 and validated in R 4.5.1 (packages survival 3.8‑3 and survRM2 1.0‑4). The follow-up duration was summarized using the reverse Kaplan–Meier method to estimate the median (with interquartile range [IQR]); the data cutoff was 31 July 2020. Categorical variables were compared using Fisher’s exact or χ² tests, as appropriate. OS was analyzed in the FAS with Kaplan–Meier curves and log‑rank tests; hazard ratios (HRs) and 95% confidence intervals (CIs) were estimated by Cox models and are reported as “Arm B vs Arm A” (HR >1 indicates a higher hazard in Arm B). The proportional‑hazards (PH) assumption was assessed by linear regression of scaled Schoenfeld residuals on log(time). To complement Cox estimates, restricted mean survival time (RMST) was calculated at prespecified horizons τ = 24 and 36 months; between‑arm differences (B − A) and 95% CIs were obtained by stratified bootstrap (2000 resamples). We also reported 24‑ and 36‑month milestone OS from Kaplan–Meier estimates with Greenwood CIs. Age‑stratified analyses (<70 vs ≥70 years) compared arms within strata and tested age × treatment interaction for heterogeneity. Prespecified subgroup analyses were also performed by primary tumor location (right vs left) and *RAS/BRAF* status; heterogeneity was assessed by treatment-by-subgroup interaction terms in Cox models. Safety analyses were performed in treated patients with evaluable adverse-event data (Arm A, *n* = 151; Arm B, *n* = 148). Unless stated otherwise, tests were two‑sided with *p* < 0.05, and no imputation was performed.

### Ethics approval and trial registration

The original C-cubed study was conducted as a prospective randomized trial and registered in the University Hospital Medical Information Network (UMIN) clinical trial registry (UMIN000015405, registered on October 14, 2014). The Institutional Review Board of Kawasaki Medical University approved the updated OS analysis as a non-interventional observational study (approval no. 3232-01). It was separately registered in the UMIN Clinical Trials Registry (UMIN000036690, registered on May 10, 2019). All participants provided written informed consent before randomization in the original C‑cubed trial. For the present OS‑update analysis, the Institutional Review Board of Kawasaki Medical University approved the use of de‑identified trial data with a waiver of additional consent (approval No. 3232‑01). All procedures were conducted in accordance with the Declaration of Helsinki and the guidelines of Good Clinical Practice.

TFS (the primary endpoint of the parent trial) and progression-free survival were analyzed and reported in the primary C‑cubed publication^14^. As the current submission focuses on updated overall survival with quality-of-life and age-stratified analyses, and no new tumor assessment data were collected after the primary analysis, PFS and TFS were not reanalyzed or re-reported; their results remain unchanged from the original publication^14^.

No independent Data and Safety Monitoring Board (DSMB) was convened owing to the use of standard‑of‑care regimens and the modest sample size. Safety monitoring was conducted by the trial steering committee and site investigators, and all serious adverse events were reported to the respective institutional review boards in accordance with the protocol.

### Role of the funding source

The funder had no role in study design, data collection, data analysis, data interpretation, or the decision to submit the manuscript. The corresponding author had full access to all the data and was responsible for the final decision to submit the manuscript for publication.

## Results

### Patient characteristics

Figure 1 shows the patient flow diagram for the FAS. Among the 300 patients included in the FAS, baseline characteristics were generally well balanced across treatment arms and age groups (<70 vs. ≥70 years), as shown in Supplementary Data 1.

### Updated OS

**Fig. 2:**
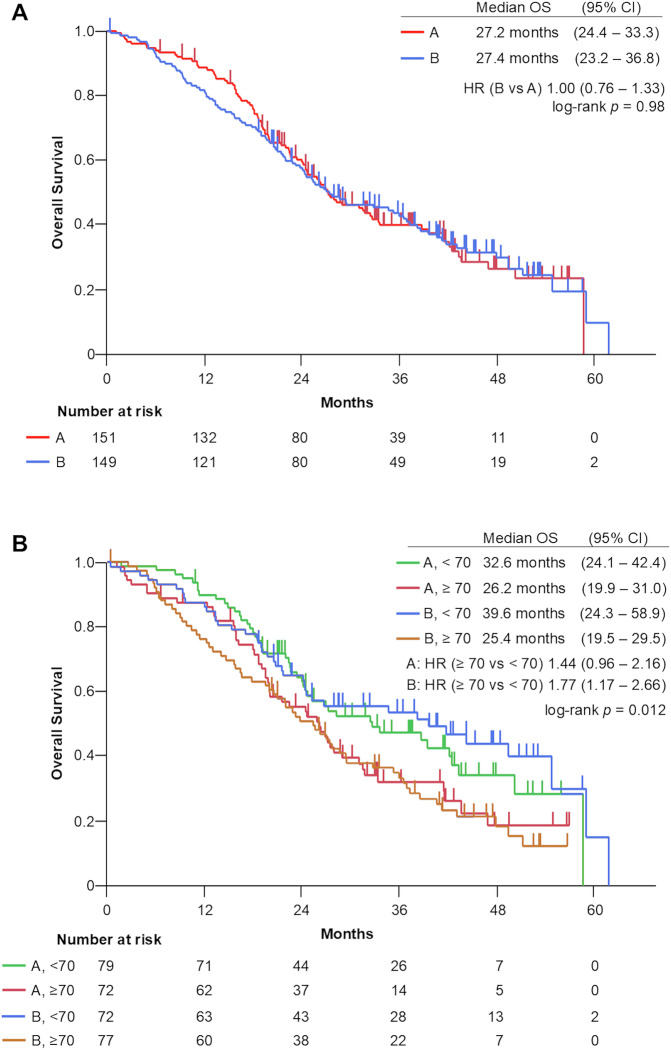
Overall survival in the C-cubed study. **A** Kaplan–Meier curves for overall survival (OS) in the full analysis set comparing sequential therapy (Arm A) and upfront oxaliplatin-based combination therapy (Arm B). Median OS and hazard ratio (HR) with 95% confidence interval (CI) are shown. HR is expressed as B versus A. The log-rank test was used for between-group comparison. Tick marks indicate censored observations. **B** Kaplan–Meier curves for OS stratified by age (<70 years vs ≥70 years) within each treatment arm. Hazard ratios compare ≥70 versus <70 years within each arm. The log-rank test was used to assess age-stratified differences. Tick marks indicate censored observations. Numbers at risk are shown below each panel.

Median follow-up was 42.7 months (IQR, 33.1–52.4) as determined by the reverse Kaplan–Meier method at the data cutoff date (31 July 2020). In the FAS, median OS was 27.2 months in Arm A (95% CI [24.4–33.3 months]) and 27.4 months in Arm B (95% CI [23.2–36.8 months]). No statistically significant difference was detected between strategies (HR [B vs. A] = 1.00, 95% CI [0.76–1.33] Wald *p* = 0.98). Kaplan–Meier curves suggested early separation in favor of Arm A (Fig. 2A), prompting prespecified PH checks and complementary RMST/milestone analyses. The PH test did not indicate a violation overall (slope = −0.056; *p* = 0.22).

### Age-stratified analyses

Age-stratified analyses showed no significant age × treatment interaction for OS (interaction *p* = 0.45). Within each treatment arm, older patients (≥70 years) had shorter OS compared with younger patients (<70 years), with HRs of 1.44 (95% CI, 0.96–2.16) in the sequential arm and 1.77 (95% CI, 1.17–2.66) in the upfront combination arm (Fig. 2B). These findings indicate that age itself was associated with prognosis, but did not significantly modify the relative treatment effect between strategies.

### Tumor location and RAS/BRAF mutation status

Tumor biology strongly influenced survival outcomes across treatment arms. Patients with left-sided primary tumors consistently demonstrated longer OS than those with right-sided tumors (Supplementary Fig. S1). Similarly, OS differed markedly by *RAS/BRAF* mutation status, following a consistent gradient of wild-type > *RAS*-mutant > *BRAF* V600E-mutant disease (Supplementary Fig. S2). These patterns were observed irrespective of treatment strategy, underscoring the dominant prognostic role of tumor location and molecular subtype.

### RMST and milestone analyses

RMST and milestone OS analyses across prespecified time horizons (24 and 36 months) yielded results concordant with the primary OS analysis, with only small, non-significant differences between strategies across age groups, tumor location, and *RAS/BRAF* status (Supplementary Tables S1A–D and S2A–D).

### QoL (pre‑planned secondary endpoints)

**Fig. 3:**
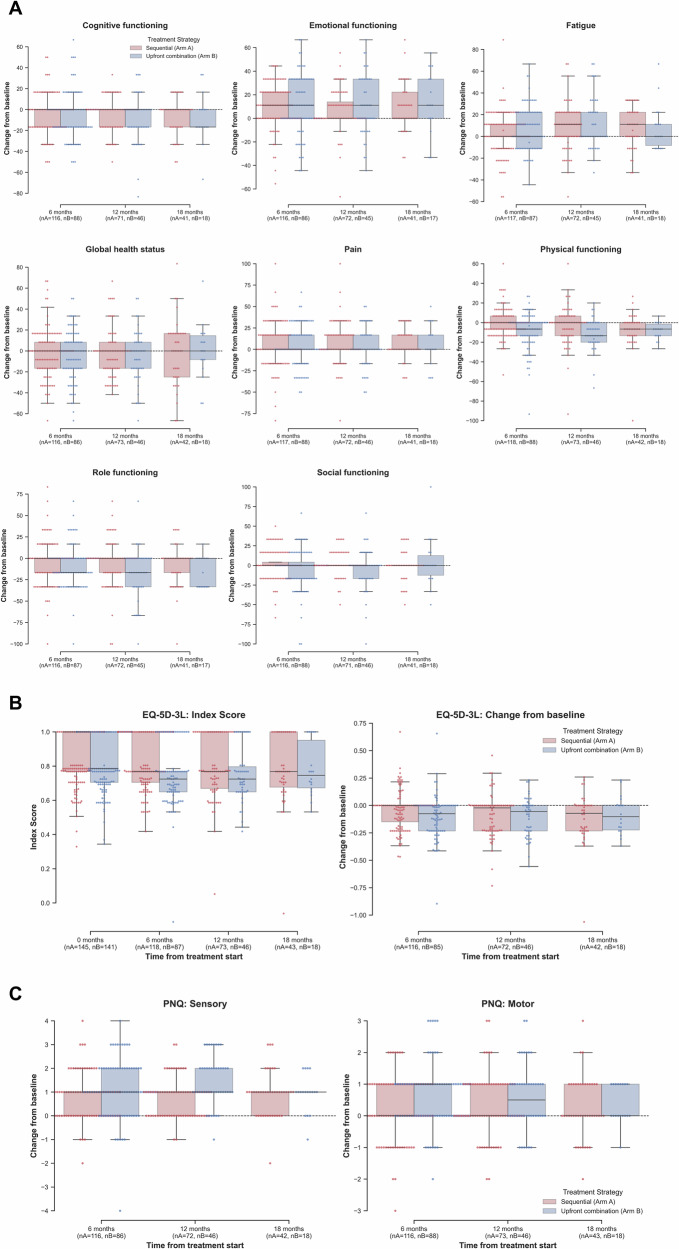
Quality-of-life and peripheral neuropathy outcomes in the C-cubed study. **A** EORTC QLQ-C30 domain scores expressed as change from baseline (Δ = follow-up − baseline) at 6, 12, and 18 months after treatment initiation. Domains shown include global health status; physical, role, emotional, cognitive, and social functioning; and symptom scales for fatigue and pain. QLQ-C30 scores were linearly transformed to a 0–100 scale according to the EORTC scoring manual. For functional scales, negative Δ values indicate deterioration; for symptom scales, positive Δ values indicate worsening. Between-arm comparisons of individual patient change scores at each time point were prespecified as exploratory and assessed using two-sided Wilcoxon rank-sum tests without multiplicity adjustment; nominal *p* values are reported in Supplementary Data 2. **B** EQ-5D-3L index utility score at baseline and at 6, 12, and 18 months (left), and change from baseline (Δ) at each follow-up time point (right). Higher index values indicate better health status; negative Δ values indicate decline from baseline. Numerical results and sample sizes are provided in Supplementary Data 2. **C** Patient Neurotoxicity Questionnaire (PNQ) sensory and motor severity shown as change from baseline (Δ) at 6, 12, and 18 months. PNQ severity was scored on a 0–4 ordinal scale (0 = none, 4 = very severe); positive Δ values indicate worsening neuropathy symptoms. Numerical results and sample sizes are provided in Supplementary Data 2.

A total of 292 patients were enrolled in the QoL substudy (Arm A, *n* = 148; Arm B, *n* = 144). Across prespecified instruments, longitudinal patterns were broadly consistent with a lower early treatment burden with the sequential strategy. In the EORTC QLQ‑C30, sequential therapy showed smaller early declines in physical (and role) functioning at 6 and 12 months, with attenuation by 18 months; changes in cognitive functioning and fatigue numerically favored Arm A but were modest and not statistically different between arms (Fig. 3A and Supplementary Data 2). EQ‑5D‑3L analyses showed a larger decline in health utility at 6 months with upfront therapy, followed by partial convergence at later time points (Fig. 3B and Supplementary Data 2). Peripheral neuropathy assessed by the PNQ increased more with upfront OX exposure at 6 and 12 months, particularly in the sensory domain, while motor symptoms remained mild; between‑arm differences narrowed by 18 months (Fig. 3C and Supplementary Data 2). All QoL comparisons were exploratory and unadjusted for multiplicity.

### Response rates

The overall response rate was higher in the upfront combination arm, consistent with the primary C-cubed report. However, response rates did not differ meaningfully by age group within each treatment arm, nor did they translate into differences in OS (Supplementary Data 3). Post-protocol subsequent treatments were administered in 106/151 patients in Arm A and 107/149 in Arm B, and the distribution of subsequent treatment categories and local therapies was broadly comparable between arms (Supplementary Table S3A). Conversion surgery/metastasectomy during the study course is summarized in Supplementary Table S3B.

### Treatment-related toxicities

Among treated patients with evaluable safety data, all-grade sensory neuropathy was more frequent with upfront OX exposure in both age strata, occurring in 80.6 versus 64.6% of patients aged <70 years and in 78.9 versus 38.9% of those aged ≥70 years (Arm B versus Arm A), whereas grade ≥3 sensory neuropathy remained uncommon (5.6 and 6.6% in Arm B among patients aged <70 and ≥70 years, respectively, versus 0% in both strata in Arm A). All-grade hematologic toxicities, particularly neutropenia, lymphopenia, and thrombocytopenia, were also numerically more frequent with upfront therapy, whereas hand-foot syndrome was more frequent with sequential treatment. This pattern was consistent with the patient-reported neuropathy findings shown in Fig. 3C. Detailed adverse-event data were provided in Supplementary Data 4.

### Prognostic factors

In univariable analyses, poorer performance status, involvement of ≥2 metastatic organs, and right-sided primary tumors were associated with inferior OS. In multivariable models restricted to baseline variables, *BRAF* V600E mutation, *RAS* mutation, PS 1, and higher metastatic burden remained independently associated with adverse outcomes, whereas treatment strategy did not (Supplementary Fig. S3 and Supplementary Table S4).

## Discussion

This updated analysis of the C‑cubed trial demonstrates that a sequential FP + BEV strategy with OX introduced at progression achieves OS comparable to upfront OX-based combination therapy, while potentially mitigating early treatment burden. No statistically significant difference in OS was observed between strategies (HR = 1.00, 95% CI [0.76–1.33]), with no evidence of violation of the PH assumption. Given the modest early separation of survival curves, we complemented Cox regression with restricted mean survival time and milestone OS analyses, which confirmed only small, non-significant time-restricted differences. These PH-robust estimators are recommended when hazards may vary over time and support clinical interpretation beyond a single HR^17,18^.

In the era of biologic therapy, randomized evidence directly comparing sequential versus upfront combination strategies remains limited. The XELAVIRI (AIO KRK-0110) study evaluated a sequential approach using an irinotecan-based backbone, whereas the present C-cubed trial specifically addressed OX-based sequencing in combination with BEV^12^. Together, these trials provide complementary evidence supporting individualized treatment sequencing in mCRC.

Consistent with prior literature, tumor-related factors—including primary tumor location and *RAS/BRAF* mutation status—were dominant determinants of prognosis, outweighing treatment sequencing itself^7,19,20^. In particular, *BRAF* V600E mutation and a higher metastatic burden emerged as the strongest adverse factors across analyses, underscoring that disease biology and extent, rather than initial chemotherapy intensity, primarily govern long-term outcomes in unselected mCRC^15,21–23^. These findings align with contemporary biomarker-driven treatment paradigms and large meta-analyses addressing sidedness and molecular subtype^9,24–27^.

Age-stratified analyses suggested differences in tolerability between strategies, with older patients experiencing greater toxicity from upfront OX exposure, particularly sensory neuropathy. Although older age was associated with shorter OS within each treatment arm, no statistically significant age × treatment interaction was observed. Together with robust patient-reported outcome data, these findings support an age- and vulnerability-adapted approach to first-line treatment selection^10,11,28^. Notably, QoL measures suggested a lower early treatment burden with sequential therapy, including smaller early declines in physical functioning and less sensory neuropathy; differences in other domains were modest.

From a broader clinical perspective, the rationale for the sequential strategy aligns closely with the principles of geriatric assessment (GA), which emphasize functional reserve, vulnerability, and QoL rather than chronological age alone^10,11^. Although formal GA was not prospectively incorporated into the C‑cubed study, the observed survival neutrality coupled with improved tolerability is consistent with a GA‑informed treatment philosophy. Specifically, the sequential approach appears well‑suited for patients with limited functional reserve or mild vulnerability identified by GA^10,11^. In such patients, aggressive upfront combination therapy may compromise functional independence and treatment continuity due to cumulative toxicity^10,11^. These risks are particularly relevant for cumulative toxicities such as oxaliplatin‑induced peripheral neurotoxicity^28^.

Based on the present findings, the sequential treatment strategy evaluated here may be clinically reasonable for patients in whom immediate maximal cytoreduction is not imperative, particularly when treatment goals prioritize preservation of function, mitigation of cumulative oxaliplatin-induced peripheral neurotoxicity, and sustained exposure to systemic therapy. This approach may be most appropriate for patients with adequate performance status and limited metastatic burden, and for those without features suggesting highly aggressive disease biology, such as *BRAF* V600E mutation. Because the present analyses did not identify a statistically significant treatment-by-subgroup interaction that would suggest a differential survival benefit from sequential treatment, this interpretation should be viewed as a clinical selection framework rather than evidence of a predictive subgroup.

Importantly, this clinical selection framework should not be directly extrapolated to biologically distinct subgroups for which alternative, biomarker-driven treatment paradigms are supported by randomized evidence. For patients with microsatellite instability-high/mismatch repair-deficient mCRC, immune checkpoint inhibition has demonstrated superior outcomes compared with chemotherapy and is widely adopted as a preferred initial approach^29^. For patients with *BRAF* V600E-mutant disease—characterized by aggressive biology—intensified cytoreductive strategies may be considered upfront (e.g., triplet chemotherapy with BEV)^22^, and *BRAF*-targeted regimens have demonstrated clinically meaningful benefit in previously treated disease (e.g., encorafenib-based therapy)^23^. Therefore, the clinical decision-making in these subgroups extends beyond the timing of OX within a BEV-based sequential strategy.

It is also important to acknowledge that contemporary standards of care for *RAS* wild-type, left-sided tumors increasingly favor EGFR-targeted therapy^9,25^. However, the C-cubed study was designed during a period when FP plus BEV was widely accepted as a backbone, particularly in strategies prioritizing tolerability and long-term treatment sustainability. This distinction highlights an important conceptual separation between biologically optimized regimens and pragmatic treatment strategies aimed at preserving QoL and continuity of care—an issue of particular relevance in older and potentially vulnerable patient populations.

In conclusion, although no statistically significant difference in OS was observed between sequential and upfront OX-based strategies, integrating time-window survival metrics and patient-reported outcomes supports the sequential approach as a rational, patient-centered option for selected individuals. Future trials incorporating formal GA may further refine patient selection and optimize treatment sequencing, advancing precision oncology not only at the molecular level but also at the level of patient vulnerability and functional reserve.

## Supplementary information

Transparent Peer Review file

Supplementary Material

Description of Additional Supplementary Files

Supplementary Data 1. Baseline characteristics

Supplementary Data 2. Quality of Life (QoL) analyses

Supplementary Data 3. Tumor Response Rates

Supplementary Data 4. Treatment-Related Adverse Events

Supplementary Data 5. Source data for Figure 2

Supplementary Data 6. Source data for Figure 3

## Data Availability

De-identified individual participant data (IPD), data dictionary, full protocol, SAP, and analysis code will be available upon publication for 5 years via a controlled-access secure environment at Kawasaki Medical School. Access will be provided to qualified researchers with a sound proposal and ethics approval, where applicable; requests should be sent to takeshin@med.kawasaki-m.ac.jp and will be reviewed within 4 weeks. A Data Use Agreement will be required, and raw IPD download will not be permitted. Source data underlying Figs. 2 and 3 are provided as Supplementary Data 5 and 6.
