# Supplementary material for: Sequential versus upfront oxaliplatin-based therapy in metastatic colorectal cancer: long-term outcomes of a randomized phase 3 trial
Source: Commun Med (Lond). 2026 May 8;6:394. doi: 10.1038/s43856-026-01633-3 (PMC13369168; doi:10.1038/s43856-026-01633-3)
Supplement: Supplementary file 2 — Supplementary Material [file 43856_2026_1633_MOESM2_ESM.pdf]

Supplementary Files

Sequential versus upfront oxaliplatin-based therapy in metastatic colorectal cancer: long term outcomes of a randomized phase 3 trial

Makoto Okawaki, MD, Ph.D.[1)], Mototsugu Shimokawa, Ph.D.[2)], Ryo Inada, MD, Ph.D.[3)], Hitoshi Ojima, MD, Ph.D.[4)], Hiroaki Tanioka, MD, Ph.D.[1)], Shingo Noura, MD, Ph.D.[5)], Yoshinori Munemoto, MD, Ph.D.[6)], Keiichiro Ishibashi, MD, Ph.D.[7)], Yoshiaki Shindo, MD, Ph.D.[8)], Madoka Hamada, MD, Ph.D.[9)], Masasumi Okajima, MD, Ph.D. [10)], Yoshiyuki Yamaguchi, MD, Ph.D.[1)], Takeshi Yamada, MD, Ph.D.[11)], Yasuhiro Shimada, MD[12)], Takeshi Nagasaka, MD, Ph.D.[1)#]

These supplementary materials provide detailed statistical results and exploratory analyses that support the findings summarized in the main Results.

Supplementary Figure S1

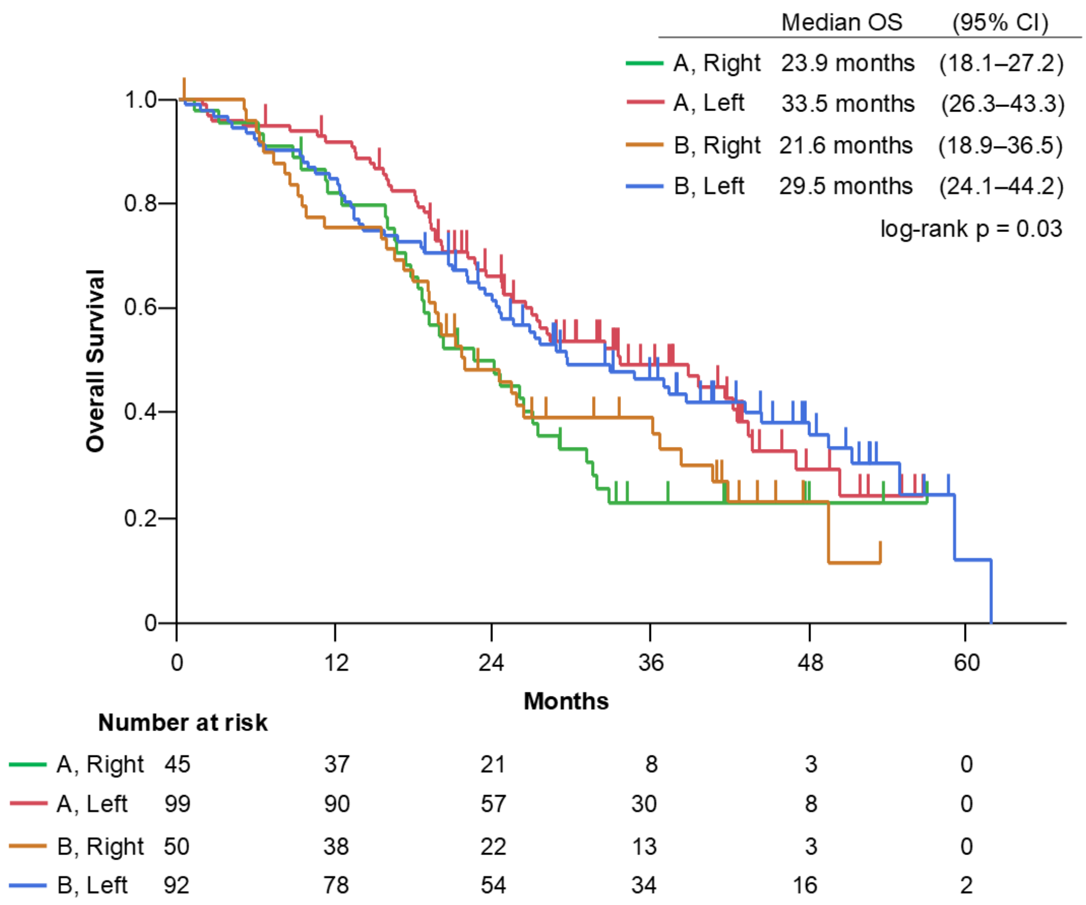

Supplementary Figure S1. Kaplan–Meier Curves for Overall Survival (OS) by primary tumor location (right vs. left colon).

P-value was derived using the log-rank test.

Supplementary Figure S2

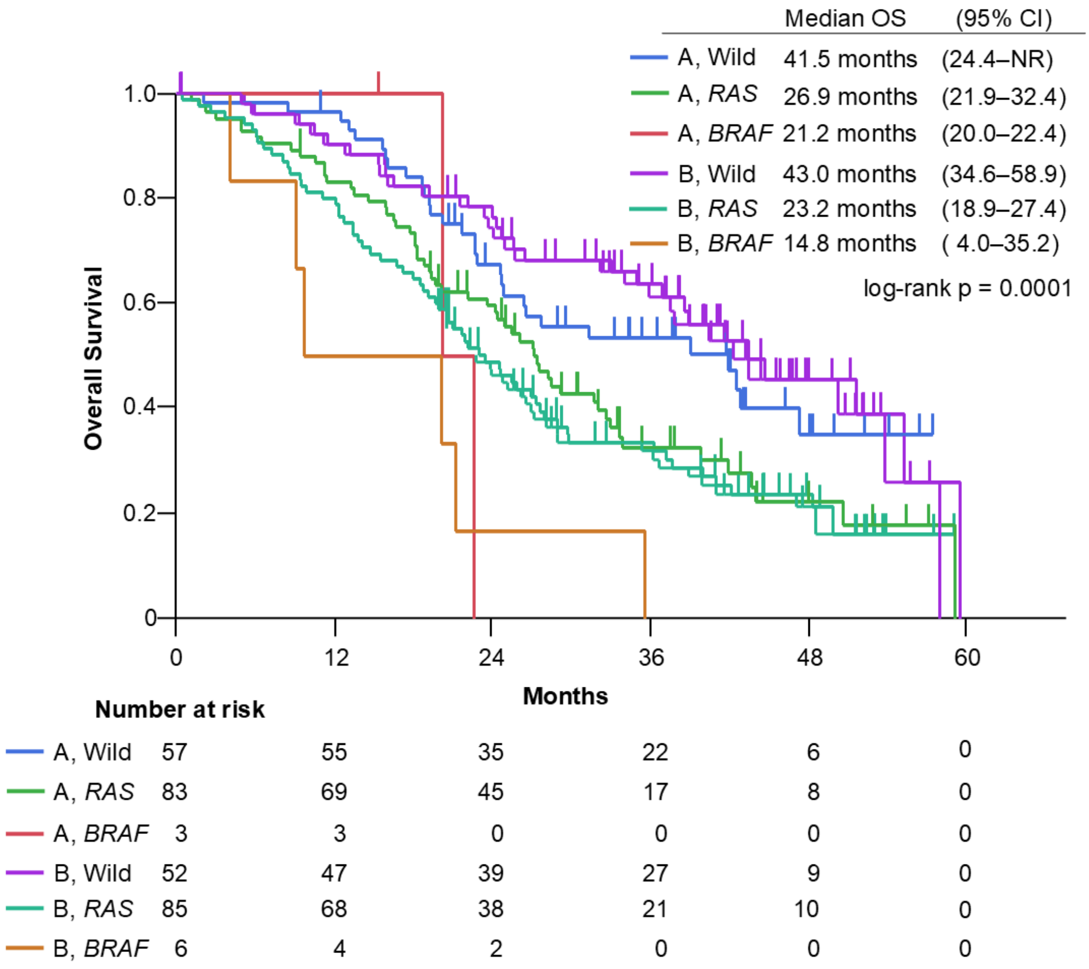

Supplementary Figure S2. Kaplan–Meier Curves for Overall Survival (OS) by *RAS/BRAF* mutation status (Wild-type, *RAS*-mutant, or *BRAF* V600E-mutant).

P-value was derived using the log-rank test. NR denotes not reached.

Supplementary Figure S3

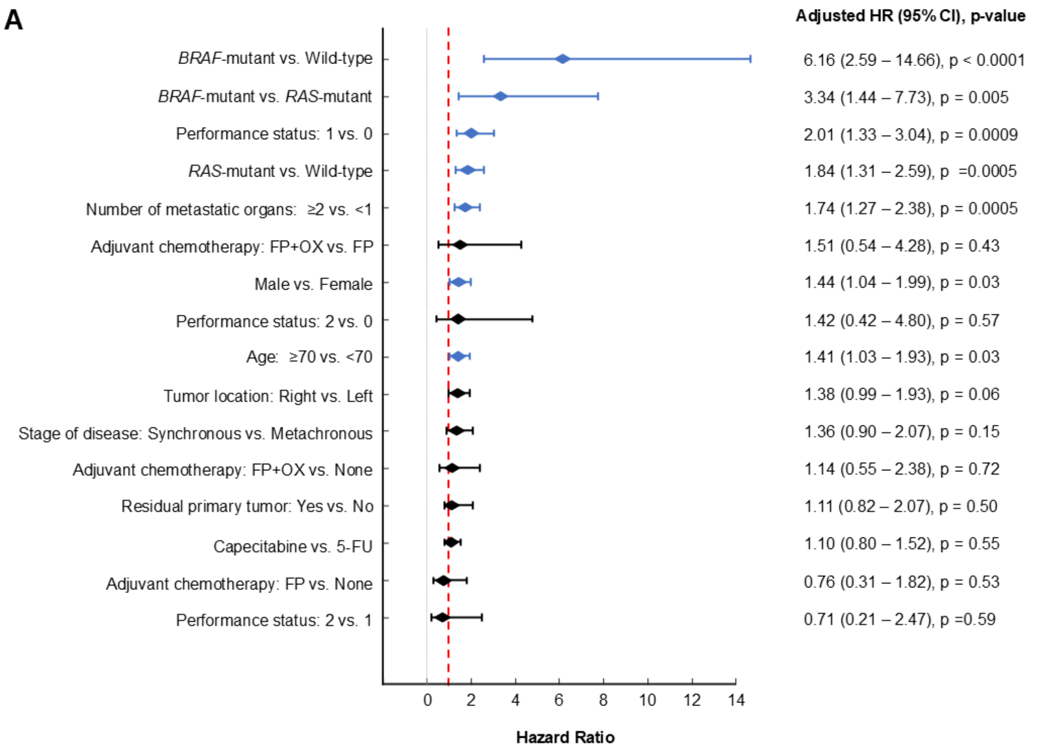

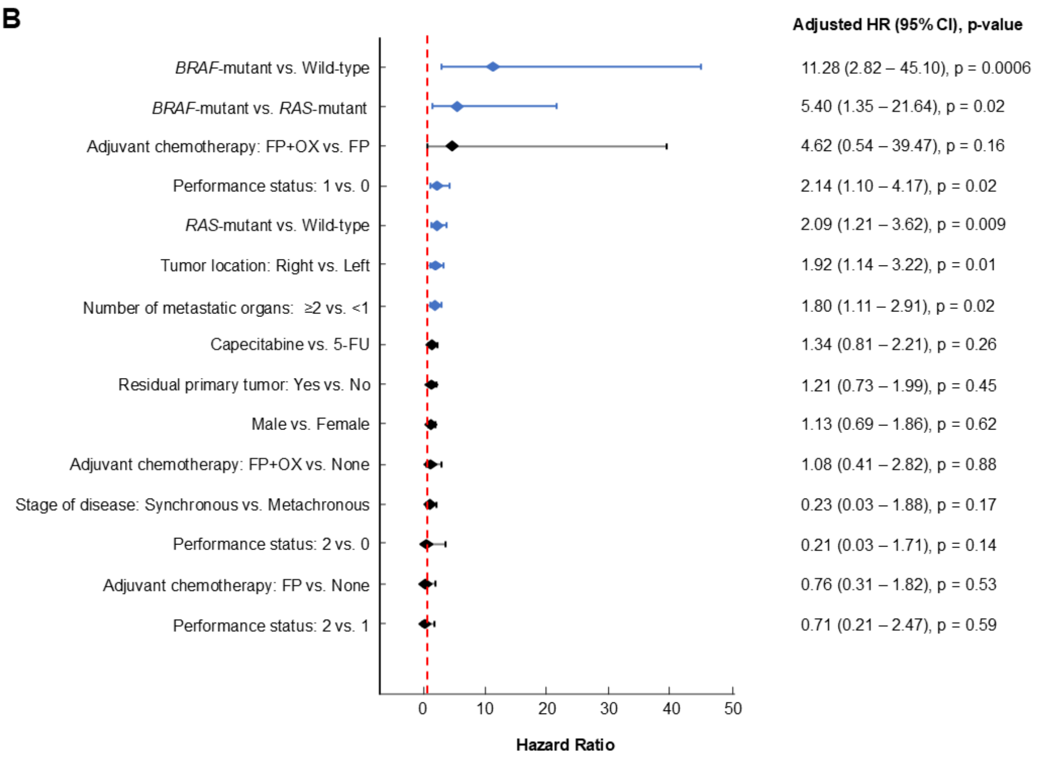

C

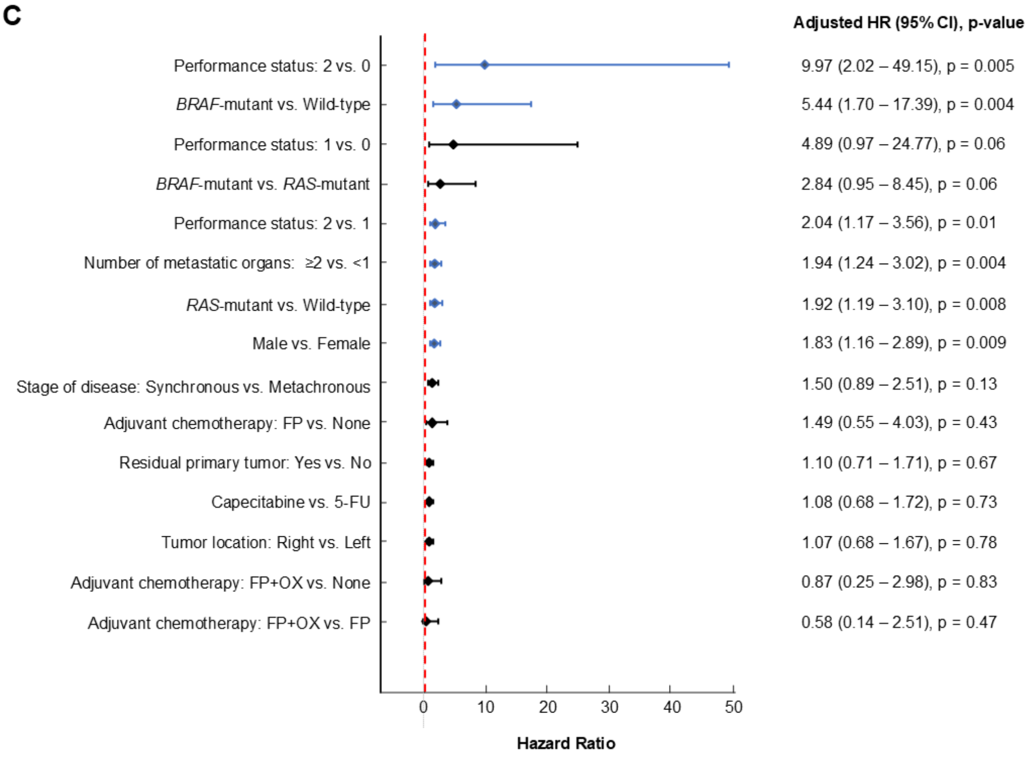

Supplementary Figure S3. Multivariable analysis of prognostic factors for overall survival (adjusted HRs) for baseline covariates.

Forest plots display adjusted hazard ratios (HRs) and 95% confidence intervals (CIs) from Cox regression models. Comparisons are oriented as indicated on the y-axis for each covariate. **(A)** Entire cohort; **(B)** < 70 years; **(C)** ≥ 70 years. Age-stratified panels are shown for completeness; no significant age × treatment interaction was observed.

Supplementary Table S1A. Restricted mean survival time (RMST) at 24 and 36 months by overall (all ages).

Cohort	Tau (months)	Arm A n (Events)	Arm A RMST, months (95% CI)	Arm B n (Events)	Arm B RMST, months (95% CI)	Δ (B − A), months (95% CI)
Overall	24	151 (94)	20.28 (19.25–21.22)	149 (99)	19.36 (18.26–20.41)	-0.92 (-2.35–0.51)
Overall	36	151 (94)	25.91 (24.16–27.59)	149 (99)	25.15 (23.22–27.00)	-0.75 (-3.24–1.63)

RMST was calculated from Kaplan–Meier estimates at prespecified horizons (τ = 24 and 36 months). Between-arm differences (B−A) and 95% confidence intervals were obtained by stratified bootstrap (2,000 resamples). Negative Δ indicates longer RMST in Arm A (sequential strategy).

Supplementary Table S1B. Restricted mean survival time (RMST) at 24 and 36 months by age group.

Cohort	Tau (months)	Arm A n (Events)	Arm A RMST, months (95% CI)	Arm B n (Events)	Arm B RMST, months (95% CI)	Δ (B − A), months (95% CI)
Age < 70	24	79 (45)	21.07 (19.90–22.11)	72 (40)	20.23 (18.78–21.60)	-0.84 (-2.60–0.96)
Age < 70	36	79 (45)	27.40 (25.28–29.53)	72 (40)	26.96 (24.34–29.43)	-0.44 (-3.83–2.96)
Age ≥ 70	24	72 (49)	19.42 (17.83–20.92)	77 (59)	18.54 (16.88–20.06)	-0.88 (-3.12–1.41)
Age ≥ 70	36	72 (49)	24.27 (21.65–26.68)	77 (59)	23.48 (20.81–26.06)	-0.79 (-4.45–2.92)

RMST was calculated from Kaplan–Meier estimates at prespecified horizons (τ = 24 and 36 months). Between-arm differences (B−A) and 95% confidence intervals were obtained by stratified bootstrap (2,000 resamples). Negative Δ indicates longer RMST in Arm A (sequential strategy).

Supplementary Table S1C. Restricted mean survival time (RMST) at 24 and 36 months by primary tumor location.

Cohort	Tau (months)	Arm A n (Events)	Arm A RMST, months (95% CI)	Arm B n (Events)	Arm B RMST, months (95% CI)	Δ (B − A), months (95% CI)
Left colon	24	99 (54)	20.97 (19.82–22.08)	92 (58)	19.71 (18.21–20.99)	-1.26 (-3.16–0.58)
Left colon	36	99 (54)	27.59 (25.45–29.68)	92 (58)	25.91 (23.47–28.19)	-1.68 (-4.88–1.48)
Right colon	24	45 (33)	18.96 (16.85–20.83)	50 (35)	18.56 (16.62–20.44)	-0.41 (-2.94–2.32)
Right colon	36	45 (33)	22.89 (19.63–25.97)	50 (35)	23.39 (20.20–26.75)	0.49 (-3.77–5.02)

RMST was calculated from Kaplan–Meier estimates at prespecified horizons (τ = 24 and 36 months). Between-arm differences (B−A) and 95% confidence intervals were obtained by stratified bootstrap (2,000 resamples). Negative Δ indicates longer RMST in Arm A (sequential strategy).

Supplementary Table S1D. Restricted mean survival time (RMST) at 24 and 36 months by *RAS/BRAF* mutation status.

Cohort	Tau (months)	Arm A n (Events)	Arm A RMST, months (95% CI)	Arm B n (Events)	Arm B RMST, months (95% CI)	Δ (B − A), months (95% CI)
Wild-type	24	57 (30)	21.60 (20.29–22.64)	52 (27)	21.63 (20.05–22.86)	0.03 (-1.82–1.80)
Wild-type	36	57 (30)	28.34 (25.76–30.74)	52 (27)	29.80 (26.99–32.39)	1.46 (-2.27–4.96)
RAS-mutant	24	83 (57)	19.58 (18.16–20.97)	85 (63)	18.45 (16.90–19.91)	-1.13 (-3.19–0.95)
RAS-mutant	36	83 (57)	24.77 (22.48–27.20)	85 (63)	22.92 (20.44–25.29)	-1.86 (-5.20–1.61)
BRAF V600E	24	3 (2)	21.21 (20.04–24.00)	6 (6)	14.58 (8.35–20.31)	-6.63 (-13.25–-0.79)
BRAF V600E	36	3 (2)	21.21 (20.04–36.00)	6 (6)	16.45 (8.45–25.74)	-4.76 (-17.94–4.16)

RMST was calculated from Kaplan–Meier estimates at prespecified horizons (τ = 24 and 36 months). Between-arm differences (B−A) and 95% confidence intervals were obtained by stratified bootstrap (2,000 resamples). Negative Δ indicates longer RMST in Arm A (sequential strategy).

Patients with unknown primary tumor location or unknown *RAS/BRAF* status were excluded from the corresponding subgroup-specific estimates. Estimates for the *BRAF* V600E subgroup are based on small sample sizes and should be interpreted cautiously. For very small subgroups, bootstrap CIs may reach the prespecified horizon (τ).

Supplementary Table S2A. Milestone overall survival rates at 24 and 36 months by overall (all ages).

Cohort	Tau (Months)	Arm A n (Events)	Arm A (95% CI)	Arm B n (Events)	Arm B (95% CI)	Δ (B − A), pp
**Overall**	24	151 (94)	59.5% (51.5–67.5%)	149 (99)	57.7% (49.7–65.7%)	-1.8 pp
	36	151 (94)	40.0% (31.6–48.4%)	149 (99)	42.8% (34.5–51.2%)	+2.8 pp

CI = confidence interval; pp = percentage points. Estimates are fixed-time Kaplan–Meier estimates with 95% CIs, as calculated by Greenwood. Differences (B − A) represent absolute percentage points calculated as Arm B minus Arm A. Values may not sum exactly due to rounding; no multiplicity adjustments were applied. These fixed-time estimates are provided for descriptive purposes and should be interpreted in conjunction with the primary OS and RMST analyses reported in the main text.

Supplementary Table S2B. Milestone overall survival rates at 24 and 36 months by age group.

Cohort	Tau (Months)	Arm A n (Events)	Arm A (95% CI)	Arm B n (Events)	Arm B (95% CI)	Δ (B − A), pp
**Age < 70**	24	79 (45)	63.0% (52.1%–74.0%)	72 (40)	64.9% (53.8%–76.0%)	1.9
**Age < 70**	36	79 (45)	47.3% (35.5%–59.2%)	72 (40)	53.7% (41.7%–65.6%)	6.3
**Age ≥ 70**	24	72 (49)	55.4% (43.7%–67.1%)	77 (59)	50.9% (39.6%–62.2%)	− 4.5
**Age ≥ 70**	36	72 (49)	32.0% (20.4%–43.7%)	77 (59)	33.4% (22.4%–44.3%)	1.3

CI = confidence interval; pp = percentage points. Estimates are fixed-time Kaplan–Meier estimates with 95% CIs, as calculated by Greenwood. Differences (B − A) represent absolute percentage points calculated as Arm B minus Arm A. Values may not sum exactly due to rounding; no multiplicity adjustments were applied. These fixed-time estimates are provided for descriptive purposes and should be interpreted in conjunction with the primary OS and RMST analyses reported in the main text.

Supplementary Table S2C. Milestone overall survival rates at 24 and 36 months by tumor location.

Cohort	Tau (Months)	Arm A n (Events)	Arm A (95% CI)	Arm B n (Events)	Arm B (95% CI)	Δ (B − A), pp
**Left colon**	24	99 (54)	66.3% (56.7%–75.8%)	92 (58)	61.6% (51.5%–71.6%)	-4.7
	36	99 (54)	49.3% (38.6%–60.1%)	92 (58)	46.7% (36.1%–57.3%)	-2.6
**Right colon**	24	45 (33)	47.7% (32.9%–62.5%)	50 (35)	48.5% (34.4%–62.6%)	0.8
	36	45 (33)	23.1% (10.2%–36.0%)	50 (35)	36.2% (22.1%–50.4%)	13.1

CI = confidence interval; pp = percentage points. Estimates are fixed-time Kaplan–Meier estimates with 95% CIs, as calculated by Greenwood. Rectal tumors were classified as left-sided per trial convention. Differences (B − A) represent absolute percentage points calculated as Arm B minus Arm A. Values may not sum exactly due to rounding; no multiplicity adjustments were applied. These fixed-time estimates are provided for descriptive purposes and should be interpreted in conjunction with the primary OS and RMST analyses reported in the main text.

Supplementary Table S2D. Milestone overall survival rates at 24 and 36 months by *RAS/BRAF* status.

Cohort	Tau (Months)	Arm A n (Events)	Arm A (95% CI)	Arm B n (Events)	Arm B (95% CI)	Δ (B − A), pp
**Wild-type**	24	57 (30)	67.4% (55.0%–79.8%)	52 (27)	76.4% (64.7%–88.1%)	9.0
	36	57 (30)	53.4% (39.9%–66.9%)	52 (27)	63.7% (50.2%–77.2%)	10.3
RAS	24	83 (57)	58.2% (47.5%–69.0%)	85 (63)	48.8% (38.0%–59.5%)	-9.4
	36	83 (57)	32.6% (21.5%–43.7%)	85 (63)	31.9% (21.3%–42.4%)	-0.7
BRAF	24	3 (2)	0.0% (0.0%–0.0%)	6 (6)	16.7% (0.0%–46.5%)	16.7
	36	3 (2)	0.0% (0.0%–0.0%)	6 (6)	0.0% (0.0%–0.0%)	0.0

CI = confidence interval; pp = percentage points. Estimates are fixed-time Kaplan–Meier estimates with 95% CIs, as calculated by Greenwood. "*BRAF*" denotes *BRAF* V600E in this dataset; subgroup estimates in *BRAF* are limited by small sample size (Arm A, n = 3; Arm B, n = 6). Differences (B − A) represent absolute percentage points calculated as Arm B minus Arm A. Values may not sum exactly due to rounding; no multiplicity adjustments were applied. These fixed-time estimates are provided for descriptive purposes and should be interpreted in conjunction with the primary OS and RMST analyses reported in the main text.

Supplementary Table S3A. Post-protocol subsequent treatments (systemic therapies and local therapies).

	Sequential treatment (Arm A)		Combination treatment (Arm B)	
**Category of subsequent therapy**	**< 70 years (n = 79)**	**≥ 70 years (n = 72)**	**< 70 years (n = 72)**	**≥ 70 years (n = 77)**
**Any subsequent therapy**	**63 (79.7)**	**43 (59.7)**	**57 (79.2)**	**50 (64.9)**
Bevacizumab-based regimens†	33 (41.8)	22 (30.6)	35 (48.6)	28 (36.4)
Anti-EGFR–based regimens‡	5 (6.3)	5 (6.9)	5 (6.9)	5 (6.5)
Ramucirumab/Aflibercept+ FOLFIRI	6 (7.6)	3 (4.2)	6 (8.3)	1 (1.3)
Regorafenib	1 (1.2)	1 (1.4)	0 (0.0)	0 (0.0)
Fluoropyrimidine alone	3 (3.8)	1 (1.4)	0 (0.0)	7 (9.1)
Oxaliplatin-based regimens	3 (3.8)	3 (4.2)	4 (5.6)	1 (1.3)
Irinotecan-based regimen	5 (6.3)	3 (4.2)	1 (1.4)	4 (5.2)
Local therapies#	6 (7.6)	4 (5.6)	6 (8.3)	4 (5.2)
Unknown	1 (1.2)	1 (1.4)	0 (0.0)	0 (0.0)

Percentages are calculated within each age stratum and treatment arm.

† Bevacizumab-based regimens include bevacizumab combined with fluoropyrimidine, irinotecan, or oxaliplatin.

‡ Anti-EGFR–based regimens include cetuximab or panitumumab with or without irinotecan or oxaliplatin.

# Local therapies include radiotherapy and/or surgery; not further classified.

Supplementary Table S3B. Conversion surgery/metastasectomy during the entire study course.

	Sequential treatment (Arm A, n = 151)		Combination treatment (Arm B, n = 149)	
	< 70 years (n = 79)	≥ 70 years (n = 72)	< 70 years (n = 72)	≥ 70 years (n = 77)
Conversion surgery/metastasectomy	7 (8.9)	5 (6.9)	8 (11.1)	1 (1.3)

Overall totals were 12/151 in Arm A and 9/149 in Arm B.
Percentages are calculated within each age stratum and treatment arm.

Supplementary Table S4. Univariable analysis of prognostic factors for overall survival (unadjusted HRs).

	All				< 70 years				≥ 70 years			
	HR	Lower 95% CI	Upper 95% CI	p-value	HR	Lower 95% CI	Upper 95% CI	p-value	HR	Lower 95% CI	Upper 95% CI	p-value
**Age: ≥70 vs. <70**	1.59	1.20	2.12	0.002	–	–	–	–	–	–	–	–
**Combination (B) vs. Sequential (A)**	1.00	0.76	1.33	0.98	0.86	0.56	1.33	0.51	1.08	0.74	1.58	0.69
**Male vs Female**	1.04	0.78	1.39	0.79	0.87	0.56	1.34	0.52	1.29	0.88	1.90	0.19
**Performance status: 2 vs. 1**	1.02	0.36	2.88	0.97	0.89	0.2	3.93	0.88	1.91	0.44	8.17	0.39
**Performance status: 2 vs. 0**	1.93	0.71	5.22	0.20	1.78	0.43	7.33	0.42	3.32	0.81	13.59	0.1
**Performance status: 1 vs. 0**	1.89	1.31	2.72	< 0.001	2.01	1.15	3.51	0.01	1.74	1.08	2.82	0.02
**Adjuvant chemotherapy: FP+OX vs. FP**	1.43	0.54	3.75	0.47	2.96	0.6	14.69	0.18	0.81	0.22	3.02	0.75
**Adjuvant chemotherapy: FP+OX vs. None**	0.72	0.38	1.37	0.32	0.81	0.35	1.86	0.62	0.73	0.27	1.98	0.53
**Adjuvant chemotherapy: FP vs. None**	0.51	0.24	1.08	0.08	0.27	0.07	1.11	0.07	0.90	0.36	2.22	0.82
**Tumor location: Right vs. Left**	1.58	1.16	2.14	0.003	1.89	1.19	3.00	0.007	1.27	0.85	1.90	0.25
**Residual primary tumor: Yes vs. No**	1.21	0.91	1.60	0.19	1.19	0.76	1.83	0.42	1.28	0.88	1.87	0.2
**Stage of disease: Synchronous vs. Metachronous**	1.33	0.96	1.85	0.09	1.50	0.87	2.6	0.14	1.33	0.87	2.01	0.18
**Number of metastatic organs: ≥2 vs. 1**	1.70	1.28	2.27	< 0.001	1.66	1.08	2.57	0.02	1.60	1.09	2.35	0.02
**Type of FP: Capecitabine vs. 5-FU**	1.20	0.90	1.60	0.2	1.38	0.89	2.12	0.15	1.08	0.74	1.59	0.68
***BRAF*-mutant vs. *RAS*-mutant**	2.17	1.05	4.46	0.04	4.69	1.44	15.23	0.01	1.42	0.57	3.56	0.45
***BRAF*-mutant vs. Wild-type**	4.06	1.92	8.60	0.0002	10.79	3.13	37.2	< 0.001	2.42	0.94	6.24	0.07
***RAS*-mutant vs. Wild-type**	1.87	1.36	2.58	0.01	2.30	1.38	3.83	0.001	1.70	1.13	2.58	0.01

Hazard ratios (HRs), 95% confidence intervals (CIs), and Wald test p-values were estimated using Cox proportional hazards models for the entire cohort as well as age-stratified subgroups (<70 years and ≥70 years). All variables were assessed using baseline data collected prior to treatment initiation.
