# Supplementary material for: Sequential versus upfront oxaliplatin-based therapy in metastatic colorectal cancer: long-term outcomes of a randomized phase 3 trial
Source: Commun Med (Lond). 2026 May 8;6:394. doi: 10.1038/s43856-026-01633-3 (PMC13369168; doi:10.1038/s43856-026-01633-3)
Supplement: Supplementary file 3 — Description of Additional Supplementary Files [file 43856_2026_1633_MOESM3_ESM.pdf]

Description of Additional Supplementary Files

File name: Supplementary Data 1

Description: Baseline characteristics

File name: Supplementary Data 2

Description: Quality of Life (QoL) analyses

File name: Supplementary Data 3

Description: Tumor Response Rates

File name: Supplementary Data 4

Description: Treatment-Related Adverse Events

File name: Supplementary Data 5

Description: Source data for Figure 2

File name: Supplementary Data 6

Description: Source data for Figure 3
