# Supplementary material for: Sequential versus upfront oxaliplatin-based therapy in metastatic colorectal cancer: long-term outcomes of a randomized phase 3 trial
Source: Commun Med (Lond). 2026 May 8;6:394. doi: 10.1038/s43856-026-01633-3 (PMC13369168; doi:10.1038/s43856-026-01633-3)
Supplement: Supplementary file 1 — Transparent Peer Review file [file 43856_2026_1633_MOESM1_ESM.pdf]

nature portfolio

Peer Review File

Sequential versus upfront oxaliplatin-based therapy in metastatic colorectal cancer: long term outcomes of a randomized phase 3 trial

Corresponding Author: Professor TAKESHI NAGASAKA

This file contains all reviewer reports in order by version, followed by all author rebuttals in order by version.

Version 0:

Reviewer comments:

Reviewer #1

(Remarks to the Author)
Okawaki and co-authors present the results of a prospective trial of considerable clinical interest which addressed the question if upfront oxaliplatin-based combination therapy with a fluoropyrimidine (FP) plus bevacizumab (BEV) is superior in time-related outcomes parameters (mainly overall survival - OS) compared to starting with a FP/BEV regimen with sequential addition oxaliplatin. There is a lot to like about the design and the analysis including the fact that patients under and over the age of 70 were looked at separately as well as the inclusion of QOL/PRO parameters.

Some comments:
- The results section is over-loaded with individual data points which truly distracts from the main message and makes this part almost not legible. I would suggest to focus on the main datapoints here and perhaps include additional information in a table
- I would be interested what the date interval of accrual to the study was. This should be included.
- The prior comment ties into the question why apparently patients with RAS/ BRAF wt cancers received a BEV containing regimen and not an EGFR mAb which is the standard of care (SOC), set by various prior trials including the Japanese PARADIGM study.
- I would like to see information of subsequent lines of therapy which of course can impact the outcome of the primary endpoint, OS.
- The authors should included data on patients who were eventually considered for surgical resection of metastases, which can occur even in patients initially not considered for metastasectomy.
- The discussion is way too short. I would like to see the authors put their findings into a clinical perspective and define the "perfect" patient who would benefit from a sequential approach. This would for instance exclude patients with a BRAF V600E mutation where the BREAKWATER data set a new SOC, as well as MSI-H/dMMR cancers where immunotherapy is established now.

Reviewer #2

(Remarks to the Author)
The authors need to be congratulated for conducting the phase-3 trial investigating whether first line FOLFOX plus Bevacizumab or a sequential strategy, starting with 5-Fu plus Bevacizumab and escalating to FOLFOX Bevacizumab at the time of progression is beneficial with respect to time to failure of strategy.
The question is relevant as side effects, especially polyneuropathy, is restricting oxaliplatin use in the majority of patients and is impacting QoL.
The manuscript is well written, the English is concise and clear. The Tables and Figures are informative and clear. However, the Forrest plots (given in the supplemental material) are difficult to interpret as it is unclear whether Arm A or Arm B is better (e.g. for females) .I therefore would recalculate the FORREST-plots comparing arm A to B as this has been your primary comparison and not (as done) e.g. male vs. female.

Version 1:

Reviewer comments:

Reviewer #1

(Remarks to the Author)
The authors have devoted considerable time and effort to accommodate my suggestions. The manuscript has clearly benefitted from these changes.

Open Access This Peer Review File is licensed under a Creative Commons Attribution 4.0 International License, which permits use, sharing, adaptation, distribution and reproduction in any medium or format, as long as you give appropriate credit to the original author(s) and the source, provide a link to the Creative Commons license, and indicate if changes were made.
In cases where reviewers are anonymous, credit should be given to 'Anonymous Referee' and the source.
The images or other third party material in this Peer Review File are included in the article’s Creative Commons license, unless indicated otherwise in a credit line to the material. If material is not included in the article’s Creative Commons license and your intended use is not permitted by statutory regulation or exceeds the permitted use, you will need to obtain permission directly from the copyright holder.
To view a copy of this license, visit https://creativecommons.org/licenses/by/4.0/

Response to Reviewers

We sincerely thank the Editor and the Reviewers for their careful and constructive evaluation of our manuscript entitled: "Sequential versus Upfront Oxaliplatin-Based Therapy in Metastatic Colorectal Cancer: Updated Overall Survival, Age-Stratified Effects, and Quality of Life from the C-cubed Study."

The insightful comments have greatly improved the manuscript's clarity, interpretability, and clinical positioning. Below, we provide a detailed point-by-point response to each comment. All revisions are highlighted in the marked-up manuscript, and detailed numerical data have been relocated to the revised Supplementary Materials where appropriate.

Reviewer #1

Comment 1

The Results section is over-loaded with individual data points, which distracts from the main message and makes this part almost not legible.

Response:

We agree with the Reviewer that the original Results section contained excessive numerical detail that obscured the main clinical message.

To address this concern, we have substantially streamlined the Results section, focusing the main text on clinically interpretable summary findings (overall survival, key subgroup patterns, and quality-of-life outcomes). Detailed numerical results—including age-stratified hazard ratios, restricted mean survival time (RMST) estimates, milestone OS values, and exploratory subgroup analyses—have been relocated to the Supplementary Materials.

This restructuring improves readability while maintaining transparency and access to all underlying data.

Location of changes:

1. Results section (multiple paragraphs revised)
2. Supplementary Tables S1–S6 and Supplementary Figures S1–S3

Comment 2

Please include the date interval of patient accrual.

Response:

We thank the Reviewer for this important suggestion. We have now explicitly stated the patient accrual period of the original C-cubed trial in the Methods section, together with clarification of the follow-up period for the OS update.

Specifically, we now indicate that patients were accrued between December 2014 and September 2016, and that follow-up for this OS update was completed at the data cutoff date of 31 July 2020.

Location of change:

1. Methods, "Study design and conduct"

Comment 3

Why did patients with RAS/BRAF wild-type tumors receive bevacizumab-based therapy rather than EGFR monoclonal antibodies, which are now standard of care?

Response:

We appreciate this comment and have clarified this point in the Discussion.

The C-cubed study was designed during a period when fluoropyrimidine plus bevacizumab was widely accepted as a backbone regimen, particularly in treatment strategies prioritizing tolerability and long-term treatment sustainability. Importantly, the primary objective of the study was not to identify the biologically most intensive first-line regimen, but to evaluate treatment sequencing and sustainability.

We now explicitly acknowledge the evolution of contemporary standards of care for RAS wild-type, left-sided tumors and discuss this issue as a contextual limitation rather than a methodological flaw.

Location of changes:

1. Discussion (paragraph addressing contemporary standards of care and EGFR-targeted therapy)

Comment 4

Please provide information on subsequent lines of therapy, which may impact overall survival.

Response:

We agree that post-protocol subsequent therapies are relevant for interpretation of OS. We have therefore summarized post-protocol subsequent treatments, defined as therapies administered after completion or failure of the assigned protocol treatment. Subsequent treatments were administered in 106/151 patients in Arm A and 107/149 in Arm B. Because post-protocol local therapy was recorded in a combined category (radiotherapy and/or surgery) and could not be reliably disaggregated, we report these as a composite category. The overall distribution of major subsequent treatment categories was broadly comparable between arms (**Supplementary Table S5A**).

Location of changes:

1. Results (brief mention)
2. Supplementary Table S5A (post-protocol subsequent treatments)

Comment 5

Please include data on patients who underwent surgical resection of metastases.

Response:

We agree that conversion surgery/metastasectomy may influence long-term survival. We have summarized the conversion surgery/metastasectomy performed during the entire study course. A total of 12 patients in Arm A and 9 patients in Arm B underwent conversion surgery/metastasectomy (**Supplementary Table S5B**). Detailed surgical procedures were not consistently captured across sites; therefore, we report this descriptively.

Location of changes:

1. Results (brief mention)
2. Supplementary Table S5B (conversion surgery/metastasectomy)

Comment 6

The Discussion is too short and should better define the “perfect” patient for a sequential strategy.

Response:

We thank the Reviewer for highlighting this key issue. The Discussion has been substantially expanded and restructured to clarify the clinical context and patient population most likely to benefit from a sequential oxaliplatin strategy.

Specifically, we:

Defined a clinical profile of patients likely to benefit (older age, preserved performance status, limited metastatic burden, microsatellite-stable tumors, non-*BRAF* V600E disease).

Explicitly stated patient populations to whom these findings should not be extrapolated (e.g., *BRAF* V600E–mutant and MSI-H/dMMR disease).

Integrated the conceptual framework of geriatric assessment (GA) to contextualize treatment sequencing without implying formal GA implementation.

Location of changes:

1. Discussion (multiple paragraphs revised and expanded)

Reviewer #2

Comment

The forest plots are difficult to interpret, as it is unclear whether Arm A or Arm B is better.

Response:

We agree with the Reviewer and have revised the forest plots to improve interpretability.

Specifically, we explicitly defined hazard ratios as Combination (Arm B) versus Sequential (Arm A), and clearly indicated the direction of effect in both the figure and legend. Under this definition, HR > 1 indicates a higher hazard with the combination strategy and therefore favors the sequential strategy (Arm A), whereas HR < 1 favors the combination strategy (Arm B).

We also added clarifying text noting that age-stratified panels are shown for completeness and that no significant age × treatment interaction was observed.

These revisions make the direction of treatment effects immediately clear.

Location of changes:

2. Supplementary Figure S3 and its legend

In Summary, in response to the Reviewers' comments, we have:

1. **Streamlined the Results section to enhance readability.**
2. **Relocated detailed numerical results to the Supplementary Materials.**
3. **Clarified the patient accrual period and OS follow-up timeframe.**
4. **Expanded the Discussion to improve clinical positioning and patient selection.**
5. **Improved the interpretability of forest plots by explicitly indicating the direction of treatment effects.**
6. **Added transparent information on post-protocol subsequent treatments and conversion surgery.**
7. **We believe that these revisions have substantially strengthened the manuscript and hope that it is now suitable for publication in Communications Medicine.**

We sincerely thank the Editor and Reviewers again for their insightful and constructive feedback.

Response to the Editor and Reviewer

Manuscript ID: COMMSMED-25-2763A

Title: Sequential versus upfront oxaliplatin-based therapy in metastatic colorectal cancer: long term outcomes of a randomized phase 3 trial

We sincerely thank you and Reviewer #1 for the careful assessment of our revised manuscript. We are very grateful for the reviewer's positive evaluation and for the recognition that the manuscript has benefited from the revisions made in response to the previous round of review.

Reviewer #1

Comment:

"The authors have devoted considerable time and effort to accommodate my suggestions. The manuscript has clearly benefitted from these changes."

Response:

We sincerely thank the reviewer for this positive and encouraging assessment. We greatly appreciate the reviewer's careful re-evaluation of our revised manuscript and the recognition that the revisions have improved the paper. No additional scientific concerns were raised in this round of review. We have also addressed the remaining editorial requests in the revised manuscript, supplementary material, and supplementary data files submitted with this revision.

We hope that the revised submission is now suitable for further consideration in ***Communications Medicine***.

Sincerely,

Takeshi Nagasaka, MD, PhD
on behalf of all authors
